# Second-Generation central venous catheter in the prevention of bloodstream infection: a systematic review**^1^**

**DOI:** 10.1590/1518-8345.0756.2722

**Published:** 2016-08-08

**Authors:** Janislei Gislei Dorociaki Stocco, Hellen Hoers, Franciele Soares Pott, Karla Crozeta, Dulce Aparecida Barbosa, Marineli Joaquim Meier

**Affiliations:** 2 RN, PhD, Hospital de Clínicas, Universidade Federal do Paraná, Curitiba, PR, Brazil.; 3 Doctoral Student, Departamento de Enfermagem, Universidade Federal do Paraná, Curitiba, PR, Brazil. Adjunct Professor, Departamento de Enfermagem, Universidade Federal do Paraná, Curitiba, PR, Brazil.; 4 Doctoral Student, Universidade Federal do Paraná, Curitiba, PR, Brasil. RN, Secretaria Estadual de Segurança Pública, Curitiba, PR, Brazil.; 5 PhD, Adjunct Professor, Departamento de Enfermagem, Universidade Federal do Paraná, Curitiba, PR, Brazil.; 6 PhD, Associate Professor, Escola Paulista de Enfermagem, Universidade Federal de São Paulo, São Paulo, SP, Brazil. 7 PhD, Associate Professor, Departamento de Enfermagem, Universidade Federal do Paraná, Curitiba, PR, Brazil.

**Keywords:** Catheterization, Central Venous, Catheter-Related Infection, Colonization, Sepsis, Meta-Analysis.

## Abstract

**Objective:**

to evaluate the effectiveness and safety in the use of second-generation central venous catheters impregnated in clorhexidine and silver sulfadiazine when compared with other catheters, being them impregnated or not, in order to prevent the bloodstream infection prevention.

**Method:**

systematic review with meta-analysis. Databases searched: MEDLINE, EMBASE, CINAHL, LILACS/SciELO, Cochrane CENTRAL; search in Congress Proceedings and records from Clinical Trials.

**Results:**

1.235 studies were identified, 97 were pre-selected and 4 were included. In catheter-related bloodstream infection, there was no statistical significance between second-generation impregnated catheter compared with the non-impregnated ones, absolute relative risk 1,5% confidence interval 95% (3%-1%), relative risk 0,68 (confidence interval 95%, 0,40-1,15) and number needed to treat 66. In the sensitivity analysis, there was less bloodstream infection in impregnated catheters (relative risk 0,50, confidence interval 95%, 0,26-0,96). Lower colonization, absolute relative risk 9,6% (confidence interval 95%, 10% to 4%), relative risk 0,51 (confidence interval 95% from 0,38-0,85) and number needed to treat 5.

**Conclusion:**

the use of second-generation catheters was effective in reducing the catheter colonization and infection when a sensitivity analysis is performed. Future clinical trials are suggested to evaluate sepsis rates, mortality and adverse effects.

## Introduction

The Central Venous Catheters (CVC) are indispensable for the treatment of critical patients both with acute and chronic illnesses. In the United States there are more than 15 million days of CVC yearly in Intensive Care Units (ICU) (meaning 15 million total days of patients exposed to CVC in the selected population)^1^. In the United Kingdom, close to 200.000 CVC are inserted every year^2^.

CVC's are beneficial to therapy, being used for specialized diagnosis and treatments, hemodynamic monitoring, parenteral nutrition, extreme osmolarity and pH fluids delivery, chemotherapy, blood and hemocomponents infusion, hemodialysis and long term antibiotic therapy. However, there are significant risks during its utilization, among them the Catheter-Related Bloodstream Infection (CRBSI) that is associated to the extended length-of-stay in up to three weeks, to morbidity, mortality and hospitalization costs^3^ ^-^ ^4^.

Several procedures are performed in order to prevent the CRBSI's such as the use of a maximal sterile barrier (cap, mask, sterile gown, gloves and sterile drapes), reduction in the time for catheter insertion, cutaneous antisepsis with clorhexidine 2% in the CVC insertion site, educational programs for the health teams, and avoiding femoral vein for the insertion^5^. Additionally it has been proposed the impregnation, coating or linkage with antimicrobials to prevent the CRBSI's^5^ ^-^ ^6^

Two types of antimicrobial agents are used to coat or impregnate the CVC - antiseptics and antibiotics. This research is focused on the second-generation CVC's impregnated in the antiseptics clorhexidine and silver sulfadiazine that have as characteristics not only the external coating but the inclusion of these antiseptics in the internal surface, the extension lines and hub^7^.

The decision to use the second-generation CVC's impregnated in clorhexidine and silver sulfadiazine is well grounded when there is a concern in expanding the prevention of CRBSI's and after the implementation of at least three basic measures to reduce infection, the training of the team that inserts and cares for the CVC, the use of maximal sterile barrier during the insertion and the cutaneous antisepsis in the insertion site of the CVC with clorhexidine at 20%^5^.

The second-generation CVC is recommended by the Centers for Disease Control and Prevention (CDC) for patients that will stay for more than five days with the device^5^ and is useful in the treatment of intensive care patients, burn patients, neutropenia patients and populations with an infection rate exceeding 3,3 per 1000 catheter/day, even when adhering to the basic preventive measures^(5,8-9)^. It is indicated in patients with previous episodes of CRBSI's and with limited options for venous access^10^, or for those with a risk factor for complications of CRBSI, such as valve or endovascular graft bearers^8^ ^,^ ^10^. 

In Brazil, second-generation, clorhexidine and silver sulfadiazine-impregnated CVC's are approved for use, by the National Agency for Health Surveillance (ANVISA) through register number 10216830036 with double and triple lumen 7 Fr. x 20 cm^11^.

The evaluation of the evidence on the effectiveness of this catheter in preventing bloodstream infection prevention is still scarce and in that sense the proposal of the present research was to search for evidence to support the decision-making process for introducing this catheter in the clinical practice.

Taking into account the specificity in the use of these devices in hospitalized patients, this research had the aim to evaluate the effectiveness and safety of the use of second-generation CVC's, impregnated in clorhexidine and silver sulfadiazine, as compared with other catheters being them impregnated or not, in the bloodstream infection prevention.

## Methods

This is a systematic review with meta-analysis, following the recommendations of the Cochrane Collaboration Handbook. To formulate the review question, a PICO^12^ strategy was used: What is the effectiveness and safety of second-generation, impregnated in clorhexidine and silver sulfadiazine CVC's, in hospitalized patients, for preventing catheter-related bloodstream infections, when compared with other catheters?

The primary outcome was to evaluate the prevention of CRBSI's and the secondary ones were the evaluation of colonization, sepsis, local infections associated to CVC or number of patients with local infections (insertion site and/or tunnel infection), adverse effects and mortality associated to bloodstream infection.

The following were considered for inclusion: randomized and quasi-randomized clinical trials, independently of publication status (published, not published, in press or ongoing), describing the use of second-generation CVC's impregnated in clorhexidine and silver sulfadiazine in hospitalized children, adolescents and adults, in the prevention or reduction of bloodstream infection, sepsis, colonization, mortality and adverse effects.

The following were excluded: studies that did not compare second-generation impregnated CVC's, duplicate and identical articles, and with other types of design other than randomized or quasi-randomized clinical trials, and studies that did not differentiate the data from adult and children patients.

The search strategy was elaborated with the help from one of the Cochrane Brazil technicians, experienced in search strategy and systematic reviews. The search was performed from January to March, 2014 and then updated in December 2014. The studies were electronically found through five databases: *Medical Literature Analysis and Retrieval System Online (*MEDLINE) (from 1948 to 2014), *Excerpta Medica Database* (EMBASE) (from 1974 to 2014), *Cumulative Index to Nursing and Health Literature* (CINAHL) (from 1982 to 2014), Literatura Latino-Americana e do Caribe em Ciências da Saúde (LILACS)/*Scientific Electronic Library Online* (SciELO) (from 1982 to 2014), Cochrane *Central Register of Controlled Trials* (CENTRAL) in The Library.

No restriction regarding language or year of publication was set forth. The search in references of systematic reviews articles and in their randomized clinical trials was also performed, as well as in registered randomized clinical trials (http://www.clinicaltrials.gov/; https://www.clinicaltrialsregister.eu/;http://www.controlled-trials.com.; http://apps.who.int/trialsearch/Default.aspx).

The search for non-published studies included checking the proceedings of congresses related to this issue (*Annals of Vascular Surgery* [1986 to 2013]; *European Society for Vascular Surgery* XXVI *Annual Meeting* [2012]; *^_14th Meeting of the European Venous Forum_^* [2013]; *European Journal of Vascular and Endovascular Surgery* [1994 to 2014]; *Journal of Vascular Nursing* [1991 to 2014] e *Journal of Vascular Surgery* [1984 to 2014]).

The search terms were the official terms and synonyms of the descriptors found in the DeCS, MeSH and EMTREE, as well as the Boolean operators AND, OR, NOT. The search strategy in MEDLINE database via PubMed was the following: ("Catheterization, Central Venous"[Mesh] OR "Catheters"[Mesh] OR Catheter* OR Vein OR Venous "Catheter-Related Infections"[Mesh] OR "Catheters, Indwelling"[Mesh]) AND "Chlorhexidine"[Mesh] OR "chlorhexidine gluconate" [Supplementary Concept] OR Chlorhexidine OR "Silver Sulfadiazine"[Mesh] OR "Silver Sulfadiazine" OR "Silver-Sulfadiazine" OR "Rifampin"[Mesh] OR Rifampi* OR "Minocycline"[Mesh] OR Minocyclin* OR "Silver iontophoretic" OR "Benzalkonium Compounds"[Mesh] OR "Benzalkonium chloride" OR "Heparin"[Mesh] OR Heparin* OR Arrowgard OR "Cook Spectrum" OR Vygon OR Vantex OR impregn* OR bond OR coat* OR "Anti-Infective Agents"[Mesh] OR antiseptic* OR antibiotic* OR antisep* OR antimicrobial). The descriptors were adapted for the rest of the analyzed databases.

Two reviewers evaluated independently the titles and abstracts of all relevant studies. They were selected using the inclusion and exclusion criteria aforementioned. When the study was relevant or if the title and abstract were inconclusive, the full text was retrieved. In case of divergence between the reviewers a third party opinion was requested, with the aim of reaching consensus.

In the case of duplicate studies, the one with more complete or recent information was included. The degree of concordance between reviewers was measured through the Kappa coefficient^13^ and the index was 0,988 with a p=<0,001, showing high concordance between the two reviewers^13^.

For the primary outcome CRBSI, were considered those patients or catheters with lab evidence of CRBSI, defined as those with isolated micro-organisms, from one or more positive blood cultures, collected separately (from peripheral blood and catheter) without any other identifiable infection source^5^ ^,^ ^14^. Other diagnostic criteria were considered as long as they were justified by valid sources.

The secondary outcomes were defined as follows: 

- Colonization - patients with CVC colonization, identified through positive culture defined with semi-quantitative culture (≥15UFC per catheter segment) positive or quantitative (≥10^2^UFC per catheter segment) proximal or distal from the catheter segment presenting the same microorganism that was isolated in the blood and in the catheter^5^ ^,^ ^14^ ^).^ Other criteria adopted by the authors of the studies were considered, as long as their definitions were justified in valid sources.

- Sepsis - patients with clinical sepsis, as diagnosed by clinical and lab criteria of the SCCM/ESICM/ACCP/ATS/SIS *International Sepsis Definitions Conference* ^15^. This definition encompasses a list of clinical characteristics for sepsis research. Other definitions adopted by authors were accepted as long as they were justified through valid sources.

- Local infections linked to CVC or number of patients with local infections (insertion site and/or tunnel infection) defined through microorganism isolation in semi-quantitative or quantitative culture in a catheter segment, with clinical signs of infection around the insertion site^(5)^.

- Adverse effects - patients that present adverse effects originated in the use of second-generation, clorhexidine and silver sulfadiazine impregnated CVC, including anaphylaxis, skin irritation and contact dermatitis.

- Mortality related to bloodstream infection, as defined through the diagnosis criteria already mentioned in the primary outcome and infection associated to decease. Other criteria adopted by the authors were also considered as long as their definitions were justified in valid sources.

Two reviewers read the selected studies and data were extracted through a pre-defined form. The information included general characteristics of the study such as outcomes, population, place and source of data, participants' inclusion and exclusion criteria, recruitment method, number of participants, loss to follow-up, information about catheter and evaluation of methodological quality.

Two reviewers, following the Cochrane Collaboration Tool for evaluation of risk of bias for randomized clinical trials, did the assessment of methodological quality^13^, making judgments dividing the studies in low, high and uncertain risk of bias in six domains: generating the randomized sequence, allocation concealment, blinding of the participants and professionals, blinding of the outcomes assessors, incomplete data on outcomes and selective reporting. Through the reviewers' judgment for each domain, the general quality of each study was deducted. Data were entered and analyzed in the program *Review Manager, version 5.3.0.*

For dichotomous variables, Relative Risk (RR), Absolute Relative Risk (ARR) and Number Needed to Treat (NNT) of beneficial outcomes with confidence interval (CI) 95% 

We performed sensitivity analyses to evaluate if the global results were affected with or without the inclusion of studies with high risk of bias, meaning those studies that were classified as with high risk of bias in any of the three key domains: randomized sequence generation, blinding and selective outcomes reporting.

The clinical heterogeneity was assessed through the type of participants, interventions and outcomes of each study. The meta-analysis was performed by outcome. The statistical heterogeneity was calculated through chi-squared test with significance level set at 10% (p<0,10)^13^. The heterogeneity test was only calculated when the meta-analysis had two or more studies and then was computed the I^2^ test. The heterogeneity was considered as important when I^2^ was larger that 50%.

The rates of CRBSI per 1.000 catheter/day were calculated using the methods as stated in chapter 9.4.8 of Cochrane Handbook ^13^. Firstly the RR was calculated by dividing the intervention group rate by the control group rate. The relative risk logarithm (log (In)) was entered in the *Review Manager*, 5.3.0 and the generic inverse variance was used.

Sensitivity analysis was performed for the primary outcome CRBSI and for the secondary ones as reported by the studies. Meta-analysis was done taking aside the low methodological quality studies as classified by the tool for assessing risk of bias in randomized controlled clinical trials.

The GRADE (*Grading of Recommendations Assessment, Development and Evaluation*) system was used to evaluate the quality of the evidences, the size of the interventions and the total of data available about the main results of the systematic review. For the statistical analysis, the GRADEpro 3.6 program was used.

We hereby declare the inexistence of funding or conflict of interest during the development of this study.

## Results

**Figure 1:**
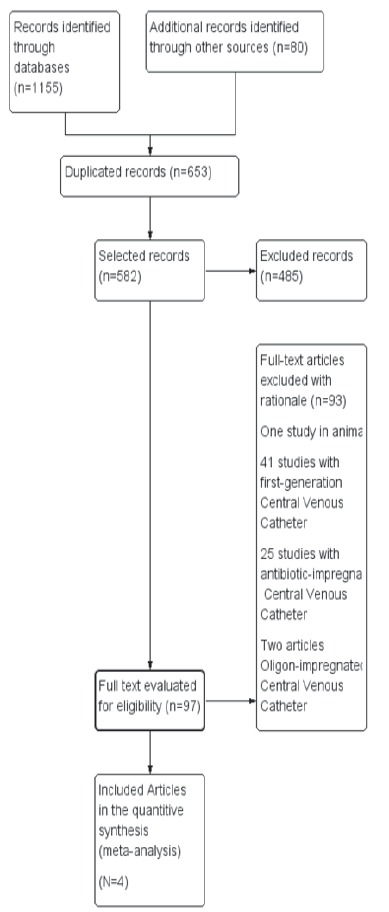
Flowchart of the process for selecting the articles for systematic review. Curitiba, PR, Brazil, 2014

Of the 1.235 studies identified, 97 were pre-selected and fully evaluated and four were included as seen in Figure 1. No studies with second-generation CVC impregnated with clorhexidine and silver sulfadiazine in children were identified. No studies in summaries of presentations in congresses or non-published studies that fulfilled the inclusion criteria were selected.

After the evaluation of the inclusion and exclusion criteria already described in the methodology, four studies were included in this review: two multi-centric, double-blind, one double-blind randomized clinical trial and one randomized clinical trial.

**Figure 2:**
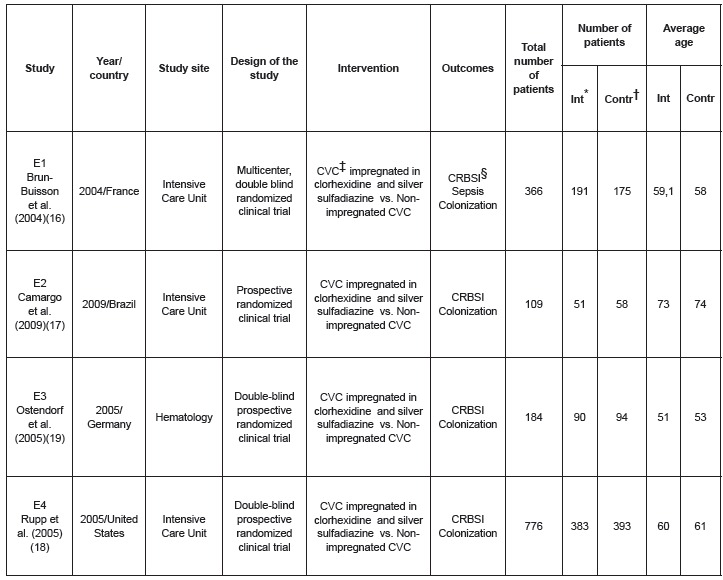
Identification of the selected studies year/country, site of the study, design, intervention, total number of patients, number of patients in intervention and control groups, average age in intervention and control groups Curitiba, PR, Brazil, 2014

The years of publication were from 2004 to 2009 and the countries of origin were France, Brazil, Germany and USA. All the interventions evaluated second-generation CVC impregnated in clorhexidine and silver sulfadiazine, compared with non-impregnated CVC; they also evaluated the primary outcome CRBSI and secondary: colonization, sepsis and local infection. The selected studies did not present secondary outcomes for adverse effects and mortality (Figure 2).

*Int – Intervention

†Contr – Control

‡CVC – Central Venous Catheter

§CRBSI – Catheter related bloodstream infection

Three studies were performed in ICU's. One of them in 14 ICU's in university hospitals in France^16^, one in a medical-surgical ICU in a private hospital in Brazil^17^ and one in ICU's of nine medical centers linked to USA universities^18^. One study^19^ addressed the cases of patients with malignant hematological disorders in a university hospital in Heidelberg, Germany.

In regard to the number of patients in the intervention and control groups, there was equivalence, being the smallest with 51 and 58 patients and the largest with 383 and 393 patients. The same applied to age. Patients in the lowest group were between 51 and 53 years old and between 73 and 74 in the highest (Figure 2).

In relation to the clinical characteristics of the patients hospitalized in the E1 study ^16^, they were clinical causes (control group 39%, intervention group 46%), scheduled surgeries (control group 13% and intervention group 10%), surgical trauma (control group 21% and intervention group 17%). Regarding the current antibiotic use, 58% were in the intervention group and 66% in the control group.

In the E2 study^17^, the diagnoses of patients admitted to ICU were linked to cardiac disorders (intervention group 4% and control group 10%), trauma (intervention group 5% and control group 3%), postoperative patients (intervention group 10% and control group 7%), respiratory failure patients (intervention group 39% and control group 30%). Antibiotic therapy was being administered in 84% of the intervention group and 79% of the control group.

In study E3^19^, patients had diagnosis of multiple myeloma (intervention group 47% and control group 42%); non-Hodgkin lymphoma (intervention group 18% and control group 15%), acute leukemia (15% in both groups). Antibiotic therapy was being administered in 6% of the intervention group and 7% of the control group.

In the E4^18^ study, the causes for admission in ICU were cardiovascular ones (intervention group 15% and control group 18%), respiratory causes (intervention group 34% and control group 38%), hematologic causes (intervention group 2% and control group 4%) and gastro-intestinal causes (intervention group 27% and control group 19%). Antibiotic therapy was being administered in 92% of the intervention group and 91% of the control group.

The evaluation of methodological quality of the four included studies for meta-analysis was performed through the Cochrane Collaboration Tool for evaluation of risk of bias for randomized clinical trials. Only two domains showed high bias risk: blinding of participants and professionals (performance bias) and incomplete outcomes data (attrition bias). 

In the first domain results happened because the study authors did not provide enough information regarding the blinding of the participants that handled the catheter (characteristic).

In the second domain, the incomplete data about losses and exclusions were the cause of the rise of the attrition bias rates and put the studies in high bias risk. There was a 50% of uncertain bias risk linked to allocation concealment, due to the lack of details about the methodology that, even though described the studies as controlled or blind, did not specified the research design data, impeding a good evaluation of the results quality.

Related to the selective outcomes reporting domain, 75% of the studies described the results of the main outcomes. For the domain Blinding of the outcomes assessors, 75% of the studies expressed how this blinding was done. In the Randomized sequence generation, only 25% of the studies were clear regarding the way this allocation sequence generation was performed.

Only the E4^18^ study showed a low bias risk in all the assessed domains, proof of a high methodological quality. In three studies there was at least two domains classified as uncertain bias risk E1^16^, E2^17^, and E3^19^ and one E2^17^ with two domains in the high bias risk classification.

**Figure 3:**
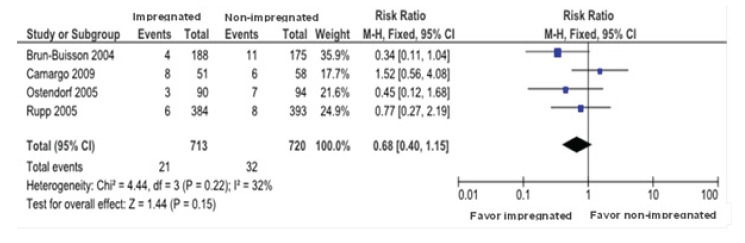
Comparison between impregnated vs. non-impregnated catheter for the catheter-related bloodstream infection. Curitiba, PR, Brazil, 2014

For the CRBSI outcome, the individuals allocated to the impregnated CVC when compared to the non-impregnated CVC group showed the following results (Figure 3).

a) Confirmed cases of CRBSI equal to 2.9%, corresponding to 21 of 713 individuals for impregnated catheters versus 4.4% (32 in 720) for non-impregnated; ARR of 1.5% (CI 95%, 3% to 1%) pointing to the benefit of the intervention; and NNT of 66 meaning that to get improvement in one patient it is needed to treat 66.

b) The global result shows that there was no statistically significant difference between the impregnated catheters compared with those that are non-impregnated for CRBSI with RR of 0.68 (CI 95%, 040-1.15). In this way, the horizontal lines that represent the CI's of the studies cross the vertical central line of the graphic (null line) as well as the diamond (lozenge) touches the null line, meaning that there is no statistical difference between intervention and control groups related to beneficial or harmful effects for CRBSI. Heterogeneity was low with I²=32%.

The evaluation of CRBSI per 1000 catheter/day was done in three studies E1(16), E2(17) and E4(19), totaling 1.179 patients, not showing statistical differences between the impregnated and not-impregnated CVC with RR of 0.94 (CI 95%, 0.45-1.95). There was no heterogeneity among studies with I²=0%.

For sensitivity analysis the reference used was the evaluation of the methodological quality according with the tool for evaluation of risk of bias for randomized clinical trials, excluding the E2 study(17) that presented the main bias risk.

**Figure 4:**
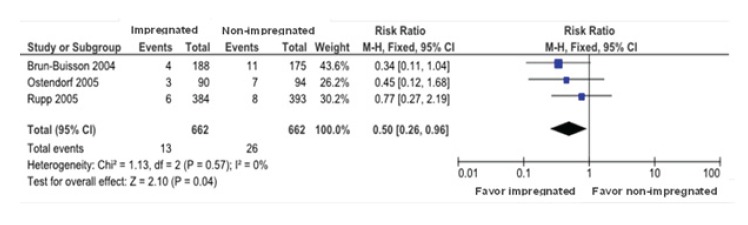
Sensitivity analysis for comparing impregnated vs non-impregnated for the outcome catheter-related bloodstream infection. Curitiba, PR, Brazil, 2014

The CRBSI showed a significant reduction in the impregnated vs. the non-impregnated catheters with a RR of 0.50 (CI 95%, 0.26-0.96), meaning that the three studies cross the null line represented by the CI, and exceed the 1 value, showing that the effect may be due to chance, however, the diamond is not touching the null line showing that there is a reduction on the risk of CRBSI for the impregnated catheters. No heterogeneity was found among the studies (I2=0) (Figure 4).

**Figure 5:**
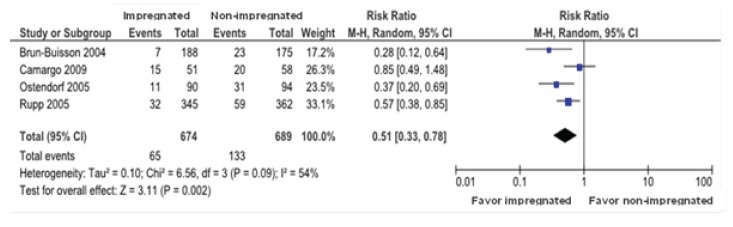
Comparison between impregnated vs. non-impregnated catheter for the Colonization outcome. Curitiba, PR, Brazil, 2014

For the outcome Colonization (Figure 5), comparing second-generation impregnated CVC vs non-impregnated CVC, the four studies were evaluated with a total of 1.363 patients, from which 674 pertained to the impregnated CVC and 689 of the non-impregnated group. The colonization of the impregnated CVC group as compared with the non-impregnated CVC showed:

a) the confirmed cases of colonization were 9.6% (65 of 674 individuals) vs. 19% (133 of 689); ARR 19% (CI 95%, 10% to 4%) showing the benefit of the intervention, the NNT of 5 points out to the fact that to get an improvement in one patient it is needed to treat 5 patients with colonization. 

b) a reduction of colonization in impregnated catheters is observed, with a RR 0.51 (CI 95% from 0.33-0.78), meaning that the horizontal lines represented by the CI's do not cross the null line, except for one study, and the diamond also does not touch the null line, showing that the impregnated catheter lowers the colonization when compared with non impregnated ones. The heterogeneity among the studies was high with I²=54%.

Three studies E1^16^, E2^17^ and E4^19^ describe the colonization per 1000 catheter/day, amounting to a total of 1.189 patients. No statistical differences were found related to benefits or harmful effects between impregnated or non-impregnated catheters with a RR of 0.69 (CI 95%, 0.47-1.01).

In the sensitivity analysis for the Colonization outcome, when the E2^17^ ^)^ is not considered, the heterogeneity was of I²=30% showing a low heterogeneity among studies. There was a decreased colonization in the impregnated catheters when compared with the non-impregnated ones with a RR of 0.45 (CI 95%, 0.33-0.62).

In studies E3^19^ and E4^18^ it was possible to run the meta-analysis for infection in the insertion site, with a total of 891 patients. No statistical difference was found between the impregnated and non-impregnated CVC's with a RR of 0.97 (CI 95%, 0.72-1.30). Heterogeneity among studies was low I²=0%.

Regarding the permanence time of the catheter, the studies E1^16^, E3^19^ e E4^18^ had that data for a total of 656 patients. There was no statistically significance for either benefit or harmful effects for the patients between the catheters, with RR of 1.05 (CI 95%, 0.68-1.62). The heterogeneity among studies was low I²=0%. The Sepsis outcome was exclusively identified in study E1^16^, reporting a patient of the intervention group that presented a septic shock one hour after the insertion of the catheter, leading to its removal following a supposition that an allergic reaction may be happening, but in the end it was attributed to sepsis.

In relation to the Adverse Events outcome, in the E4^18^ study a total of 41 (10.7%) deaths were described, due to subjacent causes in the intervention group patients, and 43 (10.9%) deaths in the control group (pneumothorax, thrombosis, hematoma, hemothorax, allergic reaction and pulmonary embolism. One patient of the control group and two of the intervention group presented allergic non-anaphylactic reaction.

In study E1^16^, a patient had pneumothorax in the intervention group; and nine had arterial puncture in the intervention group compared with two in the control group.

The studies do not show mortality associated to the catheter. In study E4^18^, the decease causes were linked to subjacent causes.

The GRADE of this meta-analysis was evaluated according to the outcomes that allowed performing meta-analysis. For the CRBSI and infection at the insertion site outcomes, the meta-analysis quality was moderate showing the fact that further research may present larger impacts on the confidence of the estimates of effect, and can modify it. For the Colonization outcome the quality was high and it is unlikely that the estimates of the effect may be modified. Regarding the time of permanence of the catheter, the quality was low, and in that way, further research may probably have a sizable effect on the confidence on the estimate and will most probably modify it.

## Discussion

No statistical differences were found for the CRBSI outcome between impregnated and non-impregnated catheters (RR 0.69, IC 95% from 0.35-1.35). Similar results were found for CRBSI per 1000 catheter/day comparing between impregnated and non-impregnated ones (RR 0.94, CI 95%, 0.45-1.95). Nonetheless, the sensitivity analysis performed in three studies E1^16^, E3^19^ and E4^18^, showed significant reduction in CRBSI in the impregnated catheters (RR 0.50, CI 95% from 0.26-0.96). This result shows that one of the studies^17^ had low methodological quality, meaning that it had a high risk of bias for data referring to the incomplete outcomes or the blinding of the participants and the team that handled the catheter. The removal of this study of the sensitivity analysis influenced the meta-analysis indexes showing the protecting effect for CRBSI of the impregnated catheter.

In order to discuss the preceding statements, we will list the results of meta-analysis that addressed the topic of this review, however they do not evaluate the studies catheter in a separate way. One meta-analysis^20^ that evaluated 19 studies with clorhexidine and silver sulfadiazine impregnated catheters, both of first and second generation, showed a reduction of CRBSI in the impregnated catheters with RR 0.73 (CI 95%, 0.57-0.94) and for CRBSI per 1.000 catheter/day RR 1.20 (CI 95%, 0.70-2.06) showing that the latter does not present an important statistical difference.

Comparing these results with the meta-analysis of the present study, we can conclude that the results are similar for CRBSI per 1.000 catheter/day but they differ for the CRBSI's.

Analogous results to the ones in this research are identified in two meta-analyses in which there were no significant statistical differences between the first and second generation CVC impregnated in clorhexidine and silver sulfadiazine when compared to the non-impregnated, for CRBSI's with RR of 0.8 (CI 95%, 0.62-1.04)^21^. A meta-analysis ^22^ evaluated different types of impregnated catheters, among them those second-generation impregnated in clorhexidine and silver sulfadiazine, identifying CRBSI with OR 0.50 (CI 95%, 0.14-1.26).

For the Colonization outcome, the second-generation clorhexidine and silver sulfadiazine impregnated catheters, when compared with the non-impregnated ones showed a significant reduction of colonization (RR 0.51, CI 95% from 0.38-0.85). These results show the effectiveness of the impregnated catheter for colonization.

This did not happen in relation to the Colonization outcome per 1.000 catheter/day (RR 0.69; CI 95% from 0.47-1.01): no statistical difference was found between the impregnated and non-impregnated catheter. This result is attributed to the small number of included studies, totaling 118 patients.

Related to this analysis focus, a meta-analysis^20^ refers that the colonization of the first and second generation, clorhexidine and silver sulfadiazine impregnated catheters was low, with RR 0.59 (IC 95% from 0.49-0.72); same happened with the colonization per 1.000 catheter/day with RR 0.53 (CI 95%, 0.28-1.02). Exception made for the colonization per 1.000 catheter/day, those data confirm the ones found in the present research.

In other studies, results found were alike to the ones in this research. In a meta-analysis^21^ that assessed first and second-generation catheters, the RR for colonization was 0.58 (CI 95% from 0.43-0.77). In another meta-analysis^22^ the colonization in the clorhexidine and silver sulfadiazine impregnated CVC's, was also decreased (OR 0.37, CI 95% from 0.17 - 0.69).

Another outcome researched in this study was the time of permanence of the catheter, evaluated in three of the studies E1^16^, E2^17^ and E3^19^, however, it did not present differences of statistical significance, due to the small number of included studies.

Infection in the insertion site was evaluated in studies E3^19^ and E4^18^, but no statistically significant difference was found, explained by the differences among the participants and the small number of included studies. Sepsis and mortality were not evaluated for the lack of available data in the selected studies. 

The adverse effects mentioned were linked to the mechanical complications (pneumothorax, hemothorax, pulmonary embolism, arterial puncture and hematoma), allergic reactions and non-anaphylactic allergic reactions. Not withstanding this fact, the allergic reactions were not described and only two studies reported those cases^16^ ^,^ ^18^. 

No studies were found that dealt with the second-generation CVC's used in the infant population; the selected studies were solely addressing adults. During the selection of the studies for this research, we found studies directed to children just with first-generation CVC's impregnated with clorhexidine and silver sulfadiazine but these studies did not report the findings for children and adults separately.

The rest of the studies were done with catheters impregnated with Minocycline and Rifampicin, silver and heparin, encompassing randomized clinical trials and cohort observational studies.

We can thus affirm that there were no evidences allowing to evaluate the effectiveness and safety of the second-generation, clorhexidine and silver sulfadiazine impregnated catheters in the onset of sepsis, mortality, collateral effects and length-of-stay.

The present review had as limitations the insufficient number of studies that did not allow the meta-analysis for the following outcomes: sepsis, mortality and adverse effects. Only the study E4^18^ fulfilled all the criteria for assessment of bias risk as evaluated by the Cochrane Collaboration tool for assessing risk of bias in randomized clinical trials, the remainder being less clear in several of the evaluated items, such as details of the methodology, blinding of the participants and the team, losses and exclusions of catheter patients. 

The scarcity of the selected studies also show the existing gap in the evidences of use of the second-generation, clorhexidine and silver sulfadiazine impregnated CVC's. In spite of this, the aforementioned studies were developed with patients in intensive care and oncology and the meta-analysis showed, as already mentioned, that the use of second-generation clorhexidine and silver sulfadiazine impregnated CVC's is superior to the non-impregnated catheter as they present lower rates of colonization. The sensitivity analysis suggest the effectiveness of impregnated CVC's in the CRBSI; following this rationale, regulatory agencies such as the National Health Surveillance Agency (ANVISA in Portuguese), HICPAC *(Healthcare Infection Control Practices Advisory Committee)* support the use of clorhexidine and silver sulfadiazine impregnated CVC's for preventing the CRBSI^5^ ^,^ ^8^.

## Conclusions

Regarding the practice implications, the selected studies that used second-generation, clorehexidine and silver sulfadiazine impregnated CVC's evidenced benefits in the reduction of catheter colonization, meanwhile there was no evidence in the reduction of sepsis, mortality and adverse effects.

Not withstanding the fact that the evidences point to the protective effect of the second-generation clorehexidine and silver sulfadiazine impregnated CVC's, there is still the need of continuing research to evaluate the effects of those results in regard to the evolution of nosocomial infections, microbiological diagnosis and control measures for hospital-acquired infections.

In spite of the recommendations that point to the need of using second-generation, clorhexidine and silver sulfadiazine impregnated catheter, caution is required when recommending them as it was not possible to evaluate the befits in relation to sepsis, mortality and adverse effects, and also the majority of assessed population were patients of Intensive Care Units.

For future studies, it is recommended: to include relevant outcomes such as sepsis evaluation, mortality and adverse effects, larger samples to minimize the error margin and expand the precision of the results, extended and better details on the blinding procedures, to implement the quality of the study and bring down the bias, to describe more clearly the characterization of the participants and the measures for controlling infection adopted in-site, and evaluate the economic impact of the catheter use.

We recommend studies with infants to determine the effectiveness of second-generation, clorhexidine and silver sulfadiazine impregnated CVC's in this population, and to estimate the CRBSI that is linked to the use of such catheter, as well as colonization, sepsis, mortality, adverse effects and costs.
